# Lifting Wavelet-Assisted EM Joint Estimation and Detection in Cooperative Spectrum Sensing

**DOI:** 10.3390/s23177428

**Published:** 2023-08-25

**Authors:** Hengyu Tian, Xu Zhao, Shiyong Chen, Yucheng Wu

**Affiliations:** 1 School of Microelectronics and Communication Engineering, Chongqing University, Chongqing 400044, China; 2 Beijing Smart-Chip Microelectronics Technology Co., Ltd., Beijing 100192, China

**Keywords:** cooperative spectrum sensing, expect the maximum algorithm, likelihood ratio test, lifting wavelet

## Abstract

Spectrum sensing in Cognitive radio (CR) is a way to improve spectrum utilization by detecting spectral holes to achieve a dynamic allocation of spectrum resources. As it is often difficult to obtain accurate wireless environment information in real-world scenarios, the detection performance is limited. Signal-to-noise ratio (SNR), noise variance, and channel prior occupancy rate are critical parameters in wireless spectrum sensing. However, obtaining these parameter values in advance is challenging in practical scenarios. A lifting wavelet-assisted Expectation-Maximization (EM) joint estimation and detection method is proposed to estimate multiple parameters and achieve full-blind detection, which uses lifting wavelet in noise variance estimation to improve detection probability and convergence speed. Moreover, a stream learning strategy is used in estimating SNR and channel prior occupancy rate to fit the scenario where the SU has mobility. The simulation results demonstrate that the proposed method can achieve comparable detection performance to the semi-blind EM method.

## 1. Introduction

With the rapid development of mobile intelligent terminals, wearable devices, and industrial monitoring in 5G applications, the amount of mobile data traffic is growing exponentially [1]. To ensure the reliability and real-time performance of user services, higher transmission rates are required to achieve real-time data interaction. The research on sixth-generation (6G) mobile communication aims to establish a powerful network to cover ground, air, and sea communications and provide a solution superior to 5G that meets the demands of high throughput, large capacity, and low latency. Cognitive radio (CR) is an effective way to improve spectrum utilization [2]. Spectrum sensing aims to achieve real-time monitoring of spectrum holes, which is a prerequisite in CR. In cognitive networks, the secondary users (Sus) can continuously monitor and opportunistically use the licensed idle frequency bands that are not being used by the primary user (PU) [3]. For the Sus, once the PU signal is detected to re-access the currently authorized frequency, the SU must immediately give up using the frequency to avoid affecting the normal communication of the PU. The shortage of spectrum resources is widespread in various communication systems, and spectrum sensing has also been found to be applied in many fields, not only in cellular communication but also, for instance, in cognitive radar systems [4,5,6,7].

Generally speaking, different detecting techniques require different amounts of wireless environment information. According to the amount of prior information required, spectrum sensing methods can be divided into un-blind detection, semi-blind detection, and full-blind detection. The likelihood ratio test (LRT) based on the Neyman-Pearson criterion is currently known as the optimal detection method, which requests a known distribution of the received signal that is generally related to the PU information, the noise variance, and channel statistic characteristics. Therefore, the LRT is an un-blind detection method [8]. Conversely, when the detection method relies only on the obtained signal samples without any prior information, it is referred to as a fully blind detection method. Cyclostationary detection utilized the periodic stationarity of communication signals to distinguish the PU signals from noise by analyzing the difference in cyclostationary spectra [9]. As long detection times and high computational complexity are required for cyclostationary detection, it is difficult to apply them in practice. Similarly, the maximum minimum eigenvalue (MME) detection also demanded a significant amount of computation as it involved matrix calculation [10,11,12].

Owing to the intricacy of the full-blind approach, several studies employed a semi-blind approach. The semi-blind detection method expects knowledge of certain parameter values such as noise variance, PU information, or channel occupancy. Energy detection (ED) is widely used in spectrum sensing due to its easy implementation. However, the detection accuracy is susceptible to noise uncertainty, as the noise variance should be known to calculate its threshold value [13]. In addition, a generalized likelihood ratio test (GLRT) has been developed to employ maximum likelihood estimation to replace the SNR of the received signal in LRT [14]. However, the detection performance of GLRT cannot be guaranteed when the channel environment changes rapidly. There were also studies to discuss the application of GLRT in multi-antenna scenarios, which involved more complex operations [15,16]. Two-step GLRT can make the calculation simple, Ref. [17] utilizes a two-step GLRT method to achieve adaptive detection under the sub-Gaussian symmetric alpha-stable sea clutter background, considering the unknown parameters of the signal complex amplitude and covariance matrix. A semi-blind detection method for estimating the SNR using the expectation maximization (EM) algorithm was derived in [18], which achieved stable estimation performance in the presence of channel variations. Nonetheless, it only considered unknown SNR and ignored scenarios where noise variance and channel occupancy rate could not be obtained.

Compared with full-blind detection, semi-blind detection has the advantage of being easy to implement and relies on the assumption of known noise variance. In practical scenarios, it is difficult for semi-blind detection methods to obtain real-time noise variance values, which makes semi-blind detection face challenges in practice where noise uncertainty exists. The semi-blind detection method with known noise variance can availably serve as a full-blind detection method in spectrum sensing when noise variance estimation is conducted. In noise variance estimation, some special channels for noise variance estimation can effectively avoid interference from the PU signal, but this estimation method sacrifices spectrum utilization [19,20]. Moreover, the noise variance was estimated by using the eigenvalues of the covariance matrix of the received signal samples, which leads to high computational complexity [21]. In [22], a noise uncertainty estimation method was proposed to improve the threshold set using the estimated noise uncertainty interval. However, due to its demand for a huge quantity of signal samples, a large detection delay is usually generated. In [23], empirical mode decomposition was used for noise variance estimation, which transformed the traditional semi-blind ED and maximum eigenvalue detection (MED) into blind detectors. In the end, wavelet decomposition was used to separate the signal from the noise. A suboptimal cooperative sensing method based on wavelet de-noising was proposed in [24]. Furthermore, the noise variance was estimated by subtracting the de-noised signal, which was reconstructed from the original received signal [25,26].

Due to the limitations of path loss and multipath fading, local spectrum sensing performance may be affected. When reusing the licensed spectrum, it is usually hoped to minimize the impact on the PU, which relies on highly reliable spectrum sensing techniques for global sensing of the wireless channel. Cooperative spectrum sensing (CSS) using spatial diversity among SUs is a good solution to achieve highly reliable spectrum sensing [27]. In CSS, there are two types of fusion criteria based on the different forms of data that local SUs upload to the fusion center (FC), which are hard fusion criteria and soft fusion criteria [28]. Hard fusion criteria represent the local decision results as 0 or 1 and upload them to the FC. The commonly used hard decision criteria include OR, AND, and K-out-of-M criteria [29]. Classic soft fusion methods include equal gain combining (EGC), selecting combining (SC), and maximum ratio combining (MRC) [30,31,32]. The weighting coefficients of MRC and SC are dependent on the SNR of the received signal for each secondary user, and the weighting coefficient of EGC is usually set to a constant value of 1. Soft fusion methods generally acquire abundant local detection information, which leads to superior performance compared to hard fusion criteria [33].

In this paper, we investigate full-blind cooperative spectrum sensing based on the classical optimal LRT. In order to eliminate the coupling between the unknown parameter estimates, we independently discuss the noise variance estimation problem and combine it with the EM estimation algorithm to achieve fully-blind detection. The main contributions are: (1) in un-blind optimal likelihood ratio detection, many parameters need to be known in advance. Traditional full-blind detection approaches are hard to use in practice as they have high computational complexity. When the parameters are unknown, the detection problem becomes a composite hypothesis. In order to achieve full-blind spectrum sensing with multiple unknown parameters, the EM algorithm is used to estimate the noise variance, SNR of the received signal, and channel prior occupancy rate. (2) Besides, a simple noise variance estimation method based on the lifting wavelet transform is used in the EM algorithm to further shorten the detection time and improve the detection performance and fitness for actual scenarios with noise uncertainty. (3) Finally, the stream learning strategy based on historical information is used in estimating SNR and the channel prior occupancy rate, making the proposed method adaptable to the scenario where the SU has mobility.

The remainder of this paper is outlined as follows: The corresponding system model and the optimal detection problem are described in Section 2. Section 3 presents the formula for multi-parameter EM estimation and detection and proposes a Lifting Wavelet-assisted EM estimation method. The simulation results under different scenarios are presented in Section 4. Finally, the work of this paper is summarized in Section 5.

## 2. System Model

**Figure 1:**
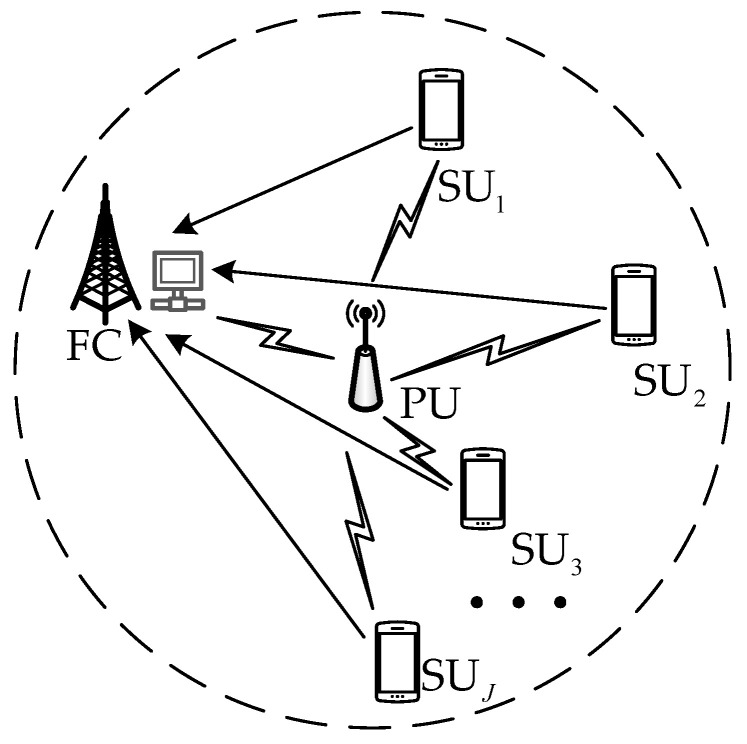
The network model of CSS.

It is assumed that the cognitive radio network consists of one PU, one FC, and *J* SUs, which is shown in Figure 1; each SU with only one antenna is not affected by interference from other SUs. Meanwhile, the reporting channel between SUs and FCs is error-free [34].

Table 1 presents the main parameters used in the analysis, along with a description of each parameter.

According to Neyman-Pearson criteria, the spectrum sensing problem can be modeled as a binary hypothesis test, and the received signal sample at the SU*_j_* is defined as [35]

(1) $$\{\begin{array}{c} y_{j}(m)=w_{j}(m),\;\;\;\;\;\;\;\;\;\;\;\;\;\;\;\;z={ℋ}_{0}\\ y_{j}(m)=h_{j}x(m)+w_{j}(m),\;\;\;\;\;z={ℋ}_{1} \end{array},$$

where z represents the channel state, $z=H_{0}$ and $z=H_{1}$ represent the absence and presence of the PU signal, respectively. The channel gain from the PU to SU*_j_* is denoted by $h_{j}$, x(m) (m=1,2,…,M) represents the *m*-th sampling point of the PU signal, which follows a Gaussian distribution with a zero mean. $w_{j}(m)$ is the zero-mean Gaussian white noise at the receiver of SU*_j_* with variance $\sigma_{j}^{2}$. It can be assumed without loss of generality that x and $w_{j}$ are independent and uncorrelated. The received signal obeys the following distribution:

(2) $$y_{j}\;\sim \;\{\begin{array}{c} N\;(0,\sigma_{j}^{2}),\;\;\;\;\;\;\;\;z={ℋ}_{0}\\ N\;(0,P{\left|h_{j}\right|}^{2}+\sigma_{j}^{2}),\;\;\;\;\;z={ℋ}_{1} \end{array}$$

where P is the transmit power of the PU signal.

To better track the parameter changes in mobile scenarios, the local detection statistics at *j*-th SU represented by $T_{j}$ is computed by using small samples, and the corresponding distribution can be described as

(3) $$T_{j}={\sum_{m=1}^{M}\left|y_{j}^{}(m)\right|}^{2}=\;\{\begin{array}{c} \sigma_{j}^{2}\chi_{M}^{2},\;\;\;\;\;\;\;\;\;\;\;\;\;\;\;\;\;z={ℋ}_{0}\\ \sigma_{j}^{2}\;(1+\gamma_{j})\chi_{M}^{2},\;\;\;\;\;\;\;z={ℋ}_{1} \end{array}$$

where the random variable $\chi_{M}^{2}$ represents a central chi-square distribution with M degrees of freedom, and $\gamma_{j}=P{\left|h_{j}\right|}^{2}/\sigma_{j}^{2}$ is the SNR of the received signal at SU*_j_*.

From (3), the probability density function (pdf) of $T_{j}$ and its logarithmic forms are:

(4) $$f_{{ℋ}_{0}}\left(T_{j}|\sigma_{j}^{2}\right)=\frac{1}{2\sigma_{j}^{2}}\frac{{\left(\frac{T_{j}}{2\sigma_{j}^{2}}\right)}^{\frac{M}{2}\;-\;1}}{\Gamma \left(\frac{M}{2}\right)\;}\;e^{-\;\frac{T_{j}}{2\sigma_{j}^{2}}}$$

(5) $$L_{{ℋ}_{0}}\left(T_{j}|\sigma_{j}^{2}\right)=(\frac{M}{2}\;-\;1)\ln(T_{j})-\frac{M}{2}\ln(2\sigma_{j}^{2})-\ln\left(\Gamma \left(\frac{M}{2}\right)\right)-\;\frac{T_{j}}{2\sigma_{j}^{2}}$$

(6) $$f_{{ℋ}_{1}}\left(T_{j}|\sigma_{j}^{2},\gamma_{j}\right)=\frac{1}{2\sigma_{j}^{2}(1+\gamma_{j})}\frac{{\left(\frac{T_{j}}{2\sigma_{j}^{2}(1+\gamma_{j})}\right)}^{\frac{M}{2}\;-\;1}}{\Gamma \left(\frac{M}{2}\right)\;}\;e^{-\;\frac{T_{j}}{2\sigma_{j}^{2}(1+\gamma_{j})}}$$

(7) $$L_{{ℋ}_{1}}\left(T_{j}|\sigma_{j}^{2},\gamma_{j}\right)=(\frac{M}{2}\;-\;1)\ln(T_{j})-\frac{M}{2}\ln(2\sigma_{j}^{2}(1+\gamma_{j}))-\ln\left(\Gamma \left(\frac{M}{2}\right)\right)-\;\frac{T_{j}}{2\sigma_{j}^{2}(1+\gamma_{j})}$$

The detection statistic, SNR, and noise variance for all SUs in the entire network are denoted as $\tilde{T}=\{T_{1},\ldots ,T_{j},\ldots ,T_{J}\}$, $\tilde{\gamma }=\{\gamma_{1},\ldots ,\gamma_{j},\ldots ,\gamma_{J}\}$, ${\tilde{\sigma }}_{}^{2}=\{\sigma_{1}^{2},\ldots ,\sigma_{j}^{2},\ldots ,\sigma_{J}^{2}\}$, respectively. The logarithmic probability density function for all SUs is the sum of each SU probability density function and can be represented as

(8) $$L_{z}(\tilde{T}|{\tilde{\sigma }}_{}^{2},\tilde{\gamma })=\log\prod_{j=1}^{J}f_{z}(T_{j}|\sigma_{j}^{2},\gamma_{j})=\sum_{j=1}^{J}L_{z}(T_{j}|\sigma_{j}^{2},\gamma_{j})$$

According to the likelihood ratio detection principle, the decision expression based on the log-likelihood ratio function for the whole work can be written as [36]

(9) $$L_{{ℋ}_{1}}(\tilde{T}|{\tilde{\sigma }}_{}^{2},\tilde{\gamma })-L_{{ℋ}_{0}}(\tilde{T}|{\tilde{\sigma }}_{}^{2})\overset{{ℋ}_{0}}{\underset{{ℋ}_{1}}{≷}}\eta ,$$

where η is the threshold value of the detection. If the difference is larger than η, it indicates the channel is occupied by the PU. Otherwise, the channel is absent and can be used by the SUs.

By substituting (5) and (7) into (9), the decision expression can be simply described as

(10) $$\sum_{j=1}^{J}\frac{\gamma_{j}}{\sigma_{j}^{2}(1+\gamma_{j})}T_{j}\underset{}{\overset{{ℋ}_{1}}{\underset{{ℋ}_{0}}{≷}}\;\mu_{LRT},}$$

where, $\mu_{LRT}=2\eta +M\sum_{j=1}^{J}\ln\left(1+\gamma_{j}\right)$ is the decision threshold, which is related to the received SNR of $\gamma_{j}$ and the threshold of η. According to (10), the global statistic is a linear combination of the detection statistic of $T_{j}$, the SNR and the noise variance.

## 3. Blind Spectrum Sensing Based on the EM Estimation

According to (10), the decision can be made if the SNR and the noise variance for each SU are known. However, these parameters usually vary in a dynamic environment. In order to determine the state of the channel, it is necessary to estimate the SNR and the noise variance of each SU. The unknown parameter vector is defined as $\theta_{u}=\{\theta ,\pi_{{ℋ}_{0}},\pi_{{ℋ}_{1}}\}$, where $\theta =\{\tilde{\gamma },{\tilde{\sigma }}_{}^{2}\}$. According to the maximum likelihood estimation theory, when the exact distribution of $T_{j}$ is known, the optimal estimation of unknown parameters could be obtained by applying the logarithmic maximum likelihood estimation, which can be represented as

(11) $$\theta_{u}^{*}=\underset{\theta_{u}}{\arg\max}\prod_{j=1}^{J}f(T_{j}|\theta_{u})=\underset{\theta_{u}}{\arg\max}\sum_{j=1}^{J}\log f(T_{j}|\theta_{u}),\;\;$$

Since the channel occupancy is unknown when SUs acquire the detection statistic of $T_{j}$, therefore z is a hidden variable during estimation. The expectation-maximization (EM) algorithm can be used to solve the maximum likelihood estimation problem with hidden variables [37]. In the EM algorithm, two steps are used to obtain the estimation parameter values, which are called the E-step and M-step, respectively. The E-step aims to obtain the conditional expectation, whereas the conditional expectation maximum is conducted to produce a new set of parameter estimates for the next round of detection in the M-step.

In the *n*-th detection, the $Q_{j,n}(z)=P\left(z={ℋ}_{z}|T_{j,n}^{},\pi_{z,n},\sigma^{2}{}_{j,n}^{},\gamma_{j,n}^{}\right)$ is defined as the posterior probability of the channel occupancy state. $Q_{j,n}({ℋ}_{1})$ represents the posterior probability of the channel being occupied based on the value of $T_{j,n}^{}$ for the *n*-th detection statistic for the *j*-th SU. According to Bayes’ rules, $Q_{j,n}(z)$ can be expressed as

(12) $$\begin{array}{c} Q_{j,n}({ℋ}_{1})=P\left(z={ℋ}_{1}|T_{j,n},\pi_{H_{1},n},\sigma^{2}{}_{j,n}^{},\gamma_{j,n}^{}\right)\\ \;\;\;\;\;\;=\frac{\pi_{{ℋ}_{1},n}^{}f_{{ℋ}_{1}}\left(T_{j,n}^{}|\sigma^{2}{}_{j,n}^{},\gamma_{j,n}^{}\right)}{\pi_{{ℋ}_{0},n}^{}f_{{ℋ}_{0}}\left(T_{j,n}|\sigma^{2}{}_{j,n}^{}\right)+\pi_{{ℋ}_{1},n}^{}f_{{ℋ}_{1}}\left(T_{j,n}|\sigma^{2}{}_{j,n}^{},\gamma_{j,n}^{}\right)}\\ \;\;\;\;\;\;\;\;\;\;\;\;\;={\left[1+\frac{\pi_{{ℋ}_{0},n}^{}}{\pi_{{ℋ}_{1},n}^{}}\frac{f_{{ℋ}_{0}}\left(T_{j,n}|\sigma^{2}{}_{j,n}^{}\right)}{f_{{ℋ}_{1}}\left(T_{j,n}|\sigma^{2}{}_{j,n}^{},\gamma_{j,n}^{}\right)}\right]}^{-1}\;\;\;\;\;\;\;\;\;\;\;\;\;\;\;\;\;\;\;\;\;\;\;\;\;\;\;\;\; \end{array}$$

(13) $$Q_{j,n}({ℋ}_{0})=1-Q_{j,n}({ℋ}_{1})$$

where $\pi_{{ℋ}_{0}}=P(z={ℋ}_{0})$ and $\pi_{{ℋ}_{1}}=P(z={ℋ}_{1})$ are channel prior occupancy rate.

(14) $$f(T_{j,n}|\gamma_{j,n},\sigma_{j,n}^{2})=\pi_{{ℋ}_{0},n}f_{{ℋ}_{0}}(T_{j,n}|\sigma_{j,n}^{2})+\pi_{{ℋ}_{1},n}f_{{ℋ}_{1}}(T_{j,n}|\gamma_{j,n},\sigma_{j,n}^{2})$$

which can be seen as the marginal distribution of $T_{j,n}^{}$ with respect to z.

The conditional expectation of (8) given the posterior probability of the channel occupancy state of $Q_{j,n-1}(z)$ is denoted as

(15) $$\begin{array}{l} \Lambda \left(\theta_{n},\pi_{z,n}\right)=E\left[\sum_{j=1}^{J}L_{z}(T_{j,n}|\sigma_{j,n}^{2},\gamma_{j,n})|Q_{j,n-1}(z)\right]\\ \;\;\;\;\;\;\;\;\;\;\;\;\;\;=\sum_{z={ℋ}_{0}}^{{ℋ}_{1}}\sum_{j}^{J}Q_{j,n-1}(z)\;\log\left\{\pi_{{ℋ}_{z},n}f_{{ℋ}_{z}}\left(T_{j,n}|\gamma_{j,n},\sigma_{j,n}^{2}\right)\right\}\\ \;\;\;\;\;\;\;\;\;\;\;\;\;\;\;\;\;\;\;\;=\sum_{j}^{J}Q_{j,n-1}^{}({ℋ}_{0})\log\left(\pi_{{ℋ}_{0},n}f_{{ℋ}_{0}}\left(T_{j,n}|\sigma_{j,n}^{2}\right)\right)\;+\;\sum_{j}^{J}Q_{j,n-1}^{}({ℋ}_{1})\log\left(\pi_{{ℋ}_{1},n}f_{ℋ1}\left(T_{j,n}|\gamma_{j,n},\sigma_{j,n}^{2}\right)\right) \end{array}$$

In the M-step, the corresponding estimation parameters of $\theta_{n}=\{{\tilde{\gamma }}_{n},{\tilde{\sigma }}_{n}^{2}\}$, $\pi_{{ℋ}_{0},n}$, and $\pi_{{ℋ}_{1},n}$ in *n*-th round of detection are obtained by maximizing the $\Lambda \left(\theta_{n},\pi_{z,n}\right)$, where the posterior probability is calculated using the estimates got from the last detection:

(16) $$Q_{j,n-1}^{}({ℋ}_{1})\approx Q_{j,n-1}^{*}({ℋ}_{1})={\left[1+\frac{\pi_{{ℋ}_{0},n-1}^{*}}{\pi_{{ℋ}_{1},n-1}^{*}}\frac{f_{{ℋ}_{0}}\left(T_{j,n-1}|\sigma^{2}{}_{j,n-1}^{*}\right)}{f_{{ℋ}_{1}}\left(T_{j,n-1}|\sigma^{2}{}_{j,n-1}^{*},\gamma_{j,n-1}^{*}\right)}\right]}^{-1}$$

(17) $$Q_{j,n-1}^{}({ℋ}_{0})\approx Q_{j,n-1}^{*}({ℋ}_{0})=1-Q_{j,n-1}^{*}({ℋ}_{1})$$

From (15), the conditional expectation $\Lambda \left(\theta_{n},\pi_{z,n}\right)$ involves optimizing multiple parameters. In order to reduce the complexity of the estimation process, when one parameter is estimated, the other parameters are assumed to have fixed values. The noise variance is estimated first by setting $\frac{\partial \Lambda \left(\theta_{n},\pi_{z,n}\right)}{\partial \sigma_{j,n}^{2}}=0$, and the estimated value is

(18) $$\sigma^{2}{}_{j,n}^{*}=\max\left(\frac{Q_{j,n-1}^{*}({ℋ}_{0})T_{j,n}+\;Q_{j,n-1}^{*}({ℋ}_{1})T_{j,n}/\left(1+\gamma_{j,n-1}^{*}\right)}{M\left(Q_{j,n-1}^{*}({ℋ}_{0})\;+\;Q_{j,n-1}^{*}({ℋ}_{1})\right)},0\right)$$

By substituting (18) into (15) and let the $\frac{\partial \Lambda \left(\theta_{n},\pi_{z,n}\right)}{\partial \gamma_{j,n}^{}}=0$:

(19) $$\gamma_{j,n}^{*}=\max\left(\frac{Q_{j,n-1}^{*}({ℋ}_{1})T_{j,n}}{MQ_{j,n-1}^{*}({ℋ}_{1})\sigma^{2}{}_{j,n}^{*}}\;-\;1,0\right)$$

Finally, we compute the $\pi_{{ℋ}_{1},n}^{*}$ and $\pi_{{ℋ}_{0},n}^{*}$ by setting $\frac{\partial \Lambda \left(\theta_{n},\pi_{z,n}\right)}{\partial \pi_{{ℋ}_{1},n}}=0$:

(20) $$\frac{\partial L\left(\theta_{n},\theta_{n-1}^{*}\right)}{\partial \pi_{{ℋ}_{1},n}}=\sum_{j=1}^{J}\frac{Q_{j,n-1}^{*}({ℋ}_{0})}{\pi_{{ℋ}_{0},n}}-\frac{Q_{j,n-1}^{*}({ℋ}_{1})}{1-\pi_{{ℋ}_{0},n}}=0$$

(21) $$\pi_{{ℋ}_{1},n}^{*}=\sum_{j=1}^{J}\frac{Q_{j,n-1}^{*}({ℋ}_{1})}{J},\;\;\;\;\pi_{{ℋ}_{0},n}^{*}=1-\pi_{{ℋ}_{1},n}^{*}$$

The detailed solution to the optimization problem is given in Appendix A.

According to the above derivation, this multi-parameter estimating method using the EM principle is denoted as a fully Blind-EM estimation and detection method.

### 3.1. Noise Variance Estimation Based on Lifting Wavelets

Because the estimated values of noise variance and SNR are coupled with each other, the performance of multi-parameter estimation will be affected in fully Blind-EM estimation and detection. Consequently, we consider decoupling the two estimates. Since the noise variance in the received signal is mainly concentrated in the high-frequency part and the wavelet transform can realize the variable resolution decomposition of the signal, the noise variance value can be estimated by using the high-frequency coefficient decomposed by the wavelet transform.

The dynamic environment caused by SU mobility puts forward a higher demand for detection delay; a shorter detection delay will help the fusion center estimate and track the changes in various parameters promptly. The traditional wavelet transform relies on the Fourier transform and has complex operations, so it is not suitable for dynamic scenarios. The lifting wavelet can realize the specific wavelet function by designing different prediction operators and update operators, which has the advantages of fast decomposition speed and small memory consumption and is completely equivalent to the traditional Mallat algorithm [38]. A low-complexity noise variance estimation method based on lifting wavelets can play a role in the EM blind estimation and detection method to enhance the performance and accelerate the convergence speed of the algorithm.

**Figure 2:**
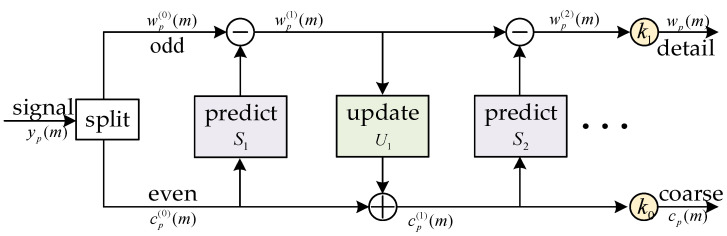
Lifting Wavelet decomposition chart.

The decomposition process of the *p*-th layer is shown in Figure 2. The lifting wavelet mainly uses prediction and update to decompose the original signal. $w_{p}^{}(m)$ and $c_{p}^{}(m)$ represent the high-frequency sub-band and low-frequency sub-band coefficients of lifting wavelet decomposition, respectively. First, the signal $y_{p}(m)$ is divided into even sequence $e_{p}(m)$ and odd sequence $o_{p}(m)$, where p represents the decomposition number of the layer, and then the repeated prediction and updating processes are conducted to complete the wavelet decomposition. Both $w_{p}^{}(m)$ and $c_{p}^{}(m)$ can be initialized separately using $o_{p}(m)$ and $e_{p}(m)$, respectively. In terms of prediction, $w_{p}^{}(m)$ can be predicted by using a prediction operator S and $c_{p}^{}(m)$, which can be expressed as

(22) $$w_{p}^{\left(k+1\right)}(m)=w_{p}^{\left(k\right)}(m)-S[c_{p}^{\left(k\right)}(m)]$$

which can be seen as the lifting of the high-pass sub-band using the low-pass sub-band, and *k* represents the number of prediction times.

Similarly, $c_{p}^{}(m)$ can be updated by using an update operator U and $w_{p}^{}(m)$, which can be expressed as

(23) $$c_{p}^{\left(k+1\right)}(m)=c_{p}^{\left(k\right)}(m)+U[w_{p}^{\left(k+1\right)}(m)]$$

where $c_{p}^{}(m)$ reflects a coarse version of the original signal, which can be seen as a process of lifting the low-frequency sub-band through the high-frequency sub-band.

If the case of *p* = 1 is considered, the db2 wavelet transform can be achieved by performing two predictions and one update, and the corresponding operator can be written as

(24) $$S_{1}=\sqrt{3},\;\;\;\;U_{1}=\frac{\sqrt{3}-2}{4}Z+\frac{\sqrt{3}}{4},\;\;\;\;S_{2}=-Z^{-1}$$

where Z and $Z^{-1}$ represent the left and right shifts of the signal in the time domain, respectively.

The specific calculation process is as follows:

(25) $$\{\begin{array}{l} w_{1}^{\left(1\right)}(m)=w_{1}^{\left(0\right)}(m)-\sqrt{3}\times c_{1}^{\left(0\right)}(m)\\ c_{1}^{\left(1\right)}(m)=(\sqrt{3}/4)\times w_{1}^{\left(1\right)}(m)+(\sqrt{3}/4-1/2)\times w_{1}^{\left(1\right)}(m+1)+c_{1}^{\left(0\right)}(m)\\ w_{1}^{\left(2\right)}(m)=c_{1}^{\left(1\right)}(m-1)+w_{1}^{\left(1\right)}(m) \end{array}$$

In the end, by multiplying the normalization factors by the final wavelet factorization coefficient, the following can be obtained:

(26) $$\left\{ \begin{array}{l} w_{1}(m)=k_{1}\times w_{1}^{\left(2\right)}(m)=(\sqrt{3}/\sqrt{2}-1/\sqrt{2})\times w_{1}^{\left(2\right)}(m)\;\\ c_{1}(m)\;=k_{0}\times c_{1}^{\left(2\right)}(m)\;=(\sqrt{3}/\sqrt{2}+1/\sqrt{2})\times c_{1}^{\left(2\right)}(m)\; \end{array}\right.$$

The noise variance can be estimated using the high-frequency coefficients obtained from the current period’s decomposition [25].

(27) $${\hat{\sigma }}_{j,n}^{2*}=\frac{median(\left|w_{1}(m)\right|)}{0.6745}$$

where $median(w_{1}(m))$ indicates the median value of the $w_{1}(m)$.

The noise variance estimation conducted in the Blind-EM algorithm is replaced by the estimation through the lifting wavelet, which is called the lifting wavelet-assisted EM iteration joint estimation method (WEMJD), which performs estimation of other unknown parameters by setting $\sigma_{j,n}^{2*}={\hat{\sigma }}_{j,n}^{2*}$. The new decision expression can be denoted as

(28) $$\sum_{j=1}^{J}\frac{\gamma_{j,n}^{*}}{{\hat{\sigma }}_{j,n}^{2*}(1+\gamma_{j,n}^{*})}T_{j,n}\underset{}{\overset{{ℋ}_{1}}{\underset{{ℋ}_{0}}{≷}}\;\mu_{WEMJD}}$$

where $\mu_{WEMJD}$ is the threshold value of WEMJD, which can be determined using a constant false alarm probability, is a detailed description, please refer to Section 3.3.

### 3.2. The SNR and CPOR Estimation

Compared with batch learning, which needs to wait until all the observed data are obtained, online learning is better suited for scenarios where data arrives continuously [39]. The parameters can be estimated and predicted over time using online streaming learning to enhance the tracking capability of parameter estimation by utilizing historical available information in dynamic environments. The related variables $Q_{j,n-1}^{*}(z)T_{j,n}$ and $Q_{j,n-1}^{*}(z)$ for parameter estimation in (19) and (21) can be written in the following form:

(29) $$\left(\begin{array}{c} a_{j,n-1}^{*}(z)\\ b_{j,n-1}^{*}(z) \end{array}\right)=\left(\begin{array}{c} a_{j,n-2}^{*}(z)\\ b_{j,n-2}^{*}(z) \end{array}\right)+u\left(\begin{array}{c} {\bar{a}}_{j,n-1}^{}(z)-a_{j,n-2}^{*}(z)\\ {\bar{b}}_{j,n-1}^{}(z)-b_{j,n-2}^{*}(z) \end{array}\right)$$

(30) $$\left(\begin{array}{c} {\bar{a}}_{j,n-1}^{}(z)\\ {\bar{b}}_{j,n-1}^{}(z) \end{array}\right)=\left(\begin{array}{c} Q_{j,n-1}^{*}(z)T_{j,n}\\ Q_{j,n-1}^{*}(z) \end{array}\right)$$

where u is the learning rate and $a_{j,n-1}^{*}(z)$ and $b_{j,n-1}^{*}(z)$ are the predicted values of $Q_{j,n-1}^{*}(z)T_{j,n}$ and $Q_{j,n-1}^{*}(z)$, respectively.

By replacing (19) and (21) with (29), the final estimating expression of SNR and channel prior occupancy rate can be expressed, respectively, as

(31) $$\gamma_{j,n}^{*}=\max\left(\frac{a_{j,n-1}^{*}({ℋ}_{1})}{Nb_{j.n-1}^{*}({ℋ}_{1})\sigma^{2}{}_{j,n}^{*}}\;-\;1,0\right)$$

(32) $$\pi_{{ℋ}_{1},n}^{*}=\sum_{j=1}^{J}\frac{b_{j.n-1}^{*}({ℋ}_{1})}{J},\;\;\;\;\;\pi_{{ℋ}_{0},n}^{*}=1-\pi_{{ℋ}_{1},n}^{*}$$

As the noise samples are independent of each other and do not have a correlation, it should be noted that historical learning is not suitable for predicting and tracking the irregular changes in the noise variance. Hence, only the sliding window is used to smooth the noise variance estimation results.

### 3.3. Initialization and Decision Thresholds

#### 3.3.1. Initialization

Due to the sensitivity of the EM algorithm to the initial values, reliable initial values can help the EM algorithm converge to the global maximum and greatly reduce the iteration time of the algorithm. Since the PU activity status is independent of the noise variance and SNR, it is assumed that the probability of the channel being occupied and unoccupied is equal at the initial time, therefore $\pi_{{ℋ}_{0},0}^{*}=\pi_{{ℋ}_{1},0}^{*}=0.5$ is set. For the initial noise variance estimation, we consider that FC has no prior knowledge of PU activity. The initial estimate of the noise variance can be acquired by (27). The initial value of the instantaneous SNR in the WEMJD method is consistent with the initial estimate value adopted in the GLRT algorithm, which can be obtained using maximum likelihood estimation and expressed as

(33) $$\Lambda_{GLR}=\log\left(\frac{f_{{ℋ}_{1}}\left(T_{j}|\sigma_{j,0}^{2*},\gamma_{j,0}\right)}{f_{{ℋ}_{0}}\left(T_{j}|\sigma_{j,0}^{2*}\right)}\right)=\frac{T_{j}\gamma_{j,0}}{2\sigma_{j,0}^{2*}\left(1+\gamma_{j,0}\right)}-\frac{N}{2}\ln\left(1+\gamma_{j,0}\right)$$

(34) $$\gamma_{j,0}^{*}=\underset{\gamma_{j}\geq 0}{\mathrm{argmax}}\Lambda_{GLR}=\max\left(\frac{T_{j,1}}{M\sigma_{j,0}^{2*}}-1,0\right)$$

#### 3.3.2. Threshold Calculation

To evaluate the performance of a detection method, its ability to correctly detect the presence of a target signal can be determined by using the false alarm probability and detection probability.

The ability of the detection method to correctly detect the presence of a target signal can be described by using the false alarm probability and detection probability. The detection probability $P_{d}=P({ℋ}_{1}|z={ℋ}_{1})$ is the probability of correctly detecting the existence of the target signal. The false alarm probability $P_{f}=P({ℋ}_{1}|z={ℋ}_{0})$ refers to the probability that the SU falsely claims PU activity when the channel is not occupied by the PU signal.

Based on the Neyman-Pearson criterion, the threshold value should maximize the detection probability with constraint $P_{f}=a$, which is known as constant false alarm rate (CFAR) detection. In CFAR detection, the threshold setting is related to the preset false alarm probability. The detection statistic in (10) is the positive weighted sum of chi-square random variables: $T=\sum_{j=1}^{J}\psi_{j}T_{j}$, $T_{j}\sim \chi_{M}^{2}(u_{r}^{2})$. The signal energy of $T_{j}$ here follows a non-central chi-square distribution, hence, $u_{r}^{2}=0$.

In general, the exact distribution of the weighted sum is difficult to obtain, So we approximate T by the chi-square distribution with *d* degrees of freedom forms like $R=\alpha \chi_{d}^{2}+\beta$ [40,41,42]. The first three cumulative moments of R and T is:

(35) $$K_{1}(R)=\alpha d+\beta$$

(36) $$K_{l}(R)=2^{l-1}(l-1)!\alpha^{l}d,\;l=2,3$$

(37) $$K_{1}(T)=M\sum_{j=1}^{J}\psi_{j},\;\;\;K_{2}(T)=2M\sum_{j=1}^{J}\psi_{j}^{2},\;\;\;K_{3}(T)=8M\sum_{j=1}^{J}\psi_{j}^{3}\;$$

where $\psi_{j}=\gamma_{j}^{*}/\left(1+\gamma_{j}^{*}\right)$, and Appendix B provides an explanation.

Let the first three cumulative moments of R and T be the same, it is obtained that

(38) $$\alpha =\frac{K_{3}(T)}{4K_{2}(T)},\;d=\frac{8K_{2}^{3}(T)}{K_{3}^{2}(T)},\;\beta =K_{1}(T)-\frac{2K_{2}^{2}(T)}{K_{3}(T)}$$

The approximate distribution of T is given by:

(39) $$F_{T}(t)\approx F_{R}=P(R\leq t)=P\left(\chi_{d}^{2}\leq \frac{t-\beta }{\alpha }\right)$$

The value of d in (38) is generally not an integer. The distribution of $\chi_{d}^{2}$ is actually a gamma distribution with a scale parameter of 2 and a shape parameter of d/2. Therefore, the chi-square distribution in (39) can be approximated by a gamma distribution and replaced as

(40) $$F_{T}(t)\approx F_{Ga}=P\left(X\leq \frac{t-\beta }{\alpha }\right),\;\;\;\;X∼Ga\left(\frac{d}{2},2\right)$$

Refer to Appendix C, the global detection threshold can be expressed as follows

(41) $$\mu_{WEMJD}=\alpha F_{{}_{Ga}}^{{}^{-1}}\left(1-P_{f}\right)+\beta$$

**Figure 3:**
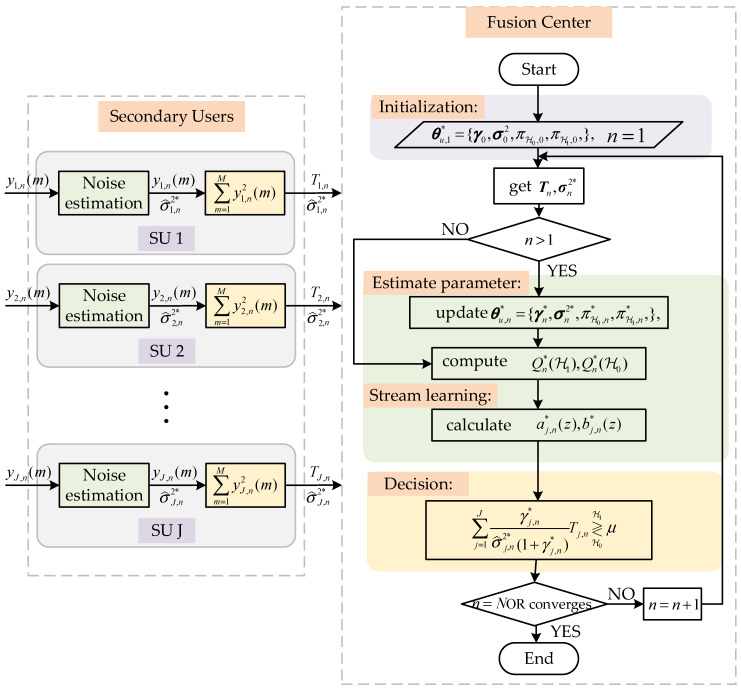
The flowchart for the proposed method at the secondary users and fusion center.

Figure 3 shows the algorithm execution process of WEMJD at the secondary users and the fusion center.

The algorithm complexity is evaluated under the case of *J* SUs, *M* sampling points, and *N* detection times. At FC, for WEMJD, Semi-blind EM and GLRT have the same computational complexity is O(J*N+M), while the complexity of matrix inversion for eigenvalue detection is $O(M^{3})$. In the case of a small sample size, *M* is the same size as *N*, while *J* is usually smaller than *M* and *N*, so the proposed method has a lower degree of complexity. At SUs, we assume that the length of the prediction operator is $l_{s}$ and the length of the update operator is $l_{u}$, and the number of decomposition layers is *p*, the complexity of the noise estimation part is $O(J*p*(l_{s}+l_{u}))$. Considering the case of p=1, the total complexity of the proposed algorithm is $O(J*N+M)+O(J*(l_{s}+l_{u}))$.

## 4. Simulation Results and Analysis

In this section, the simulation results under different settings are shown. To make the results more realistic and reliable, we performed $R=10^{4}$ Monte Carlo experiments to obtain the final results by setting the threshold based on the CFAR, where it is assumed that all SUs have the same false alarm probability of $P_{f}$.

In the network, due to the random distribution of SUs, differences in geographic location also bring about differences in received SNR. Therefore, we define the global average SNR as

(42) $$\gamma_{G}=\frac{1}{J}\sum_{j=1}^{J}\bar{\gamma_{j}}$$

where $\bar{\gamma_{j}}$ represents the average SNR at SU*_j_*.

In order to evaluate the estimation performance of the proposed algorithm, the mean squared error (MSE) is used as the evaluation metric. The MSE for the *n*-th detection is defined as

(43) $$MSE(\theta_{n}^{*})=\frac{1}{RJ}\sum_{r=1}^{R}\sum_{j=1}^{J}{\left(\theta_{j,n}^{*}-\theta_{j,n}^{}\right)}^{2}$$

### Results and Discussions

**Figure 4:**
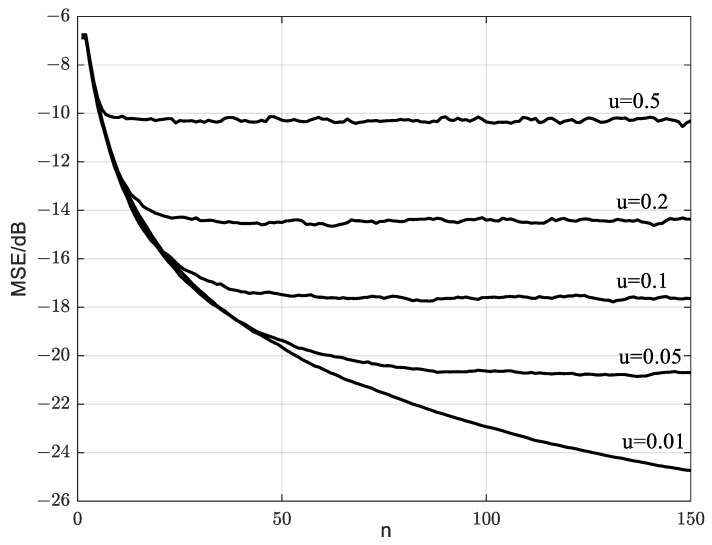
Relationship between learning rate and MSE.

To discuss the convergence of the method, it is first assumed that the secondary users are stationary and have the same nominal noise variance of $\sigma_{j}^{2}=1$. The simulation parameters are M=32, $\gamma_{G}=-3\;dB$, N=150, and $P_{f}=0.01$. Figure 4 shows the MSE for SNR estimations vary with different iteration times of n. It can be seen that the smaller MSE is obtained if the smaller u is chosen as the iteration time of n is increased. Clearly, a smaller u allows the historical data to be more thoroughly trained and it will slow down the convergence speed. In addition, since too small u will lead to over-learning of history data, it will not further enhance the detection performance by blindly reducing the value of u. Through considering the compromise between the convergence speed and the estimated performance, u=0.1 is selected for subsequent simulation.

**Figure 5:**
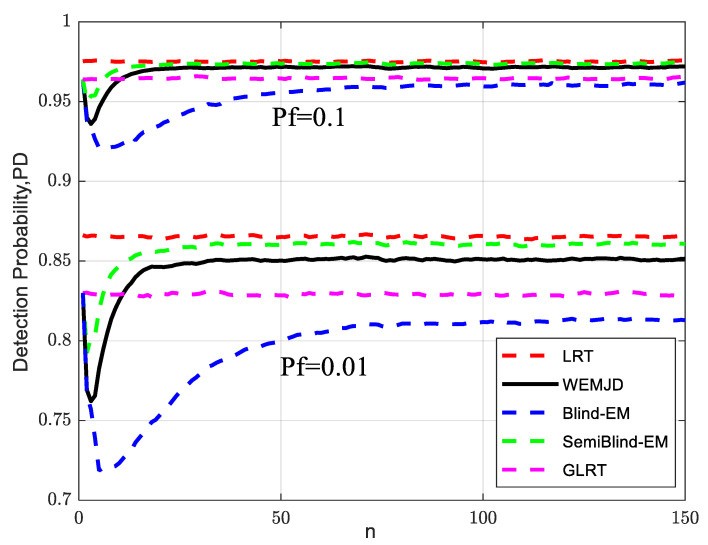
Relationship between detection probability and the number of iterations for different methods.

Figure 5 plots the relationship between detection probability and the number of iterations for different methods. The simulation parameters are set as follows M=32, $\gamma_{G}=-3\;dB$, N=150, and $P_{f}=\{0.1,0.01\}$. As the SemiBlind-EM method requires fewer joint parameter estimations compared to Blind-EM, it indicates faster method convergence. Compared with the Blind-EM which estimates all parameters by using the EM algorithm, the WEMJD algorithm exhibits a faster convergence speed as it utilizes the lifting wavelet to obtain noise variance information. Due to the limited historical information for learning in the early stages of detection, the performance of SemiBlind-EM, Blind-EM, and WEMJD methods is poor. However, as historical data accumulates, the method’s performance gradually stabilizes. It can be observed that both SemiBlind-EM and the proposed WEMJD outperform the semi-blind GLRT method, which does not incorporate learning strategies. Furthermore, the fully blind WEMJD achieves performance comparable to SemiBlind-EM while maintaining an adjacent convergence speed. Considering the convergence speed and detection performance of the method, WEMJD exhibits significant advantages, which we will mainly discuss its performance below.

The setting of the false alarm probability is related to the threshold value. From Figure 5, a lower probability of false alarms usually results in a higher threshold, which leads to the detection probability reducing with the decrease in false alarm probability. In Appendix B, during the threshold calculation, we employ the estimated value instead of the true value, which also causes the calculation of the threshold to be affected by the estimation error. So here, the threshold is related not only to the false alarm probability but also to the estimation algorithm used. The detection probability analysis reveals that the likelihood ratio test (LRT) exhibits the lowest sensitivity to false alarm probability, meaning its detection performance is less affected by changes in false alarm probability. On the other hand, the detection probability of Blind-EM, which involves the estimation of more parameters, experiences the most significant reduction as the false alarm probability increases.

**Figure 6:**
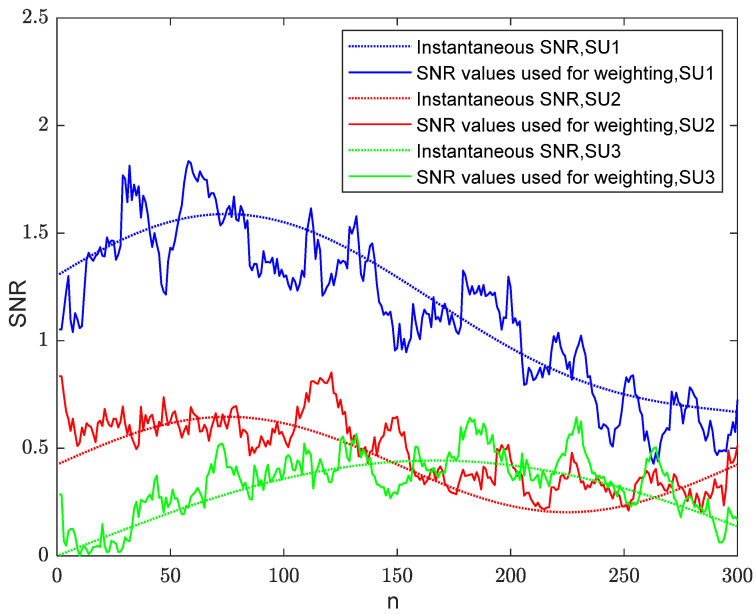
The instantaneous SNR and the estimated SNR are used for weighting at different iterations.

By taking account of the scenario of changing channel conditions, it is assumed that the SUs exhibit mobility, which means that the received SNR at the SUs changes over time. In the presence of J=3 and M=64, the estimated SNR for each user using the WEMJD method is shown in Figure 6. It can be seen that the estimated value does not conform to the change law of instantaneous SNR at the beginning. When the required number of iterations is reached, the WEMJD can effectively track and estimate the instantaneous SNR of each user’s received signal.

**Figure 7:**
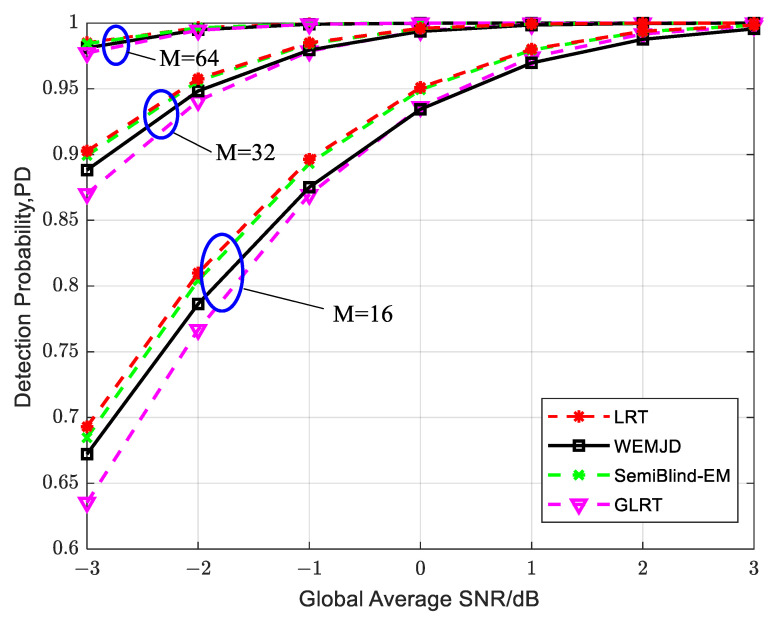
The relationship between detection probability and global average SNR under different sampling point numbers when SUs exhibit mobility.

To observe the sustained performance of the method, the simulation parameters are set as J=3, $P_{f}=0.01$, $\gamma_{G}=-3\;\mathrm{dB}$, and N=1000. As shown in Figure 7, the detection probability increases as the SNR increases. It can also be observed that the detection performance continuously improves as the sample size of M increases with the same SNR. Moreover, the difference in detection probability between the WEMJD and SemiBlind-EM gradually becomes larger as the sample size of M decreases. It can be interpreted that the estimation performance of the proposed WEMJD method will be degraded as the noise variance estimation becomes poorer as the number of sample points used becomes smaller.

**Figure 8:**
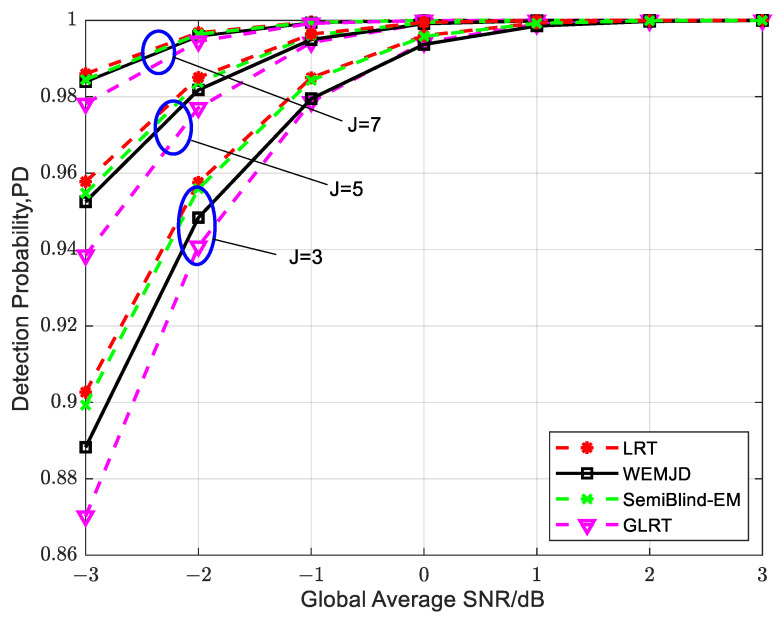
The relationship between detection probability and global average SNR under different numbers of users when SUs exhibit mobility.

Figure 8 shows the relationship between detection probability and global average SNR with different numbers of users. Obviously, as the number of users involved in cooperation increases, the detection performance improves. Moreover, with a fixed SNR, the performance improvement is more pronounced as the number of users increases from 3 to 5 compared to the increase from 5 to 7 users, which indicates that simply increasing the number of SUs in the network cannot continuously enhance the detection performance. Furthermore, the detection performance for the proposed method approximately approaches that of the optimal LRT method as more secondary users participate in spectrum sensing.

To observe the performance of different estimation and detection methods with noise uncertainty. It is assumed that the noise variance follows a uniform distribution within a certain range, $\sigma_{n}^{2}\sim U(\frac{\sigma_{n}^{2}}{u_{c}},u_{c}\sigma_{n}^{2})$ where $u_{c}$ is the fluctuation ratio, and the noise uncertainty is defined as $un=10\log_{10}(u_{c})$. In practice, it is challenging to obtain real-time accurate values of noise variance for semi-blind detection methods, while it is relatively easy to obtain the nominal power and fluctuation range of the noise variance. Therefore, the weighting coefficients are calculated by using the maximum noise variance fluctuation value $\sigma_{n\max}^{2}=u_{c}\sigma_{n}^{2}$ for the semi-blind method to minimize the impact on PU.

**Figure 9:**
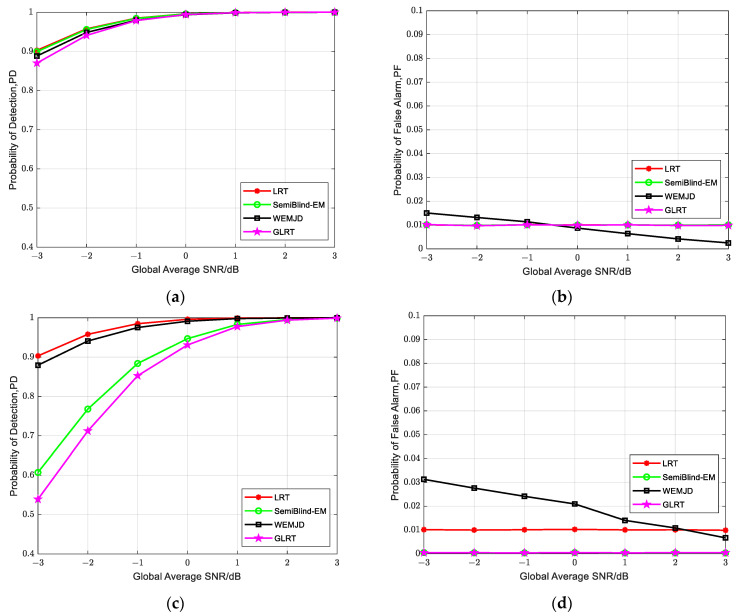
Relationship among the detection probability, the false alarm probability, and the SNR under noise uncertainty. (**a**) Detection probability under *un* = 0 dB; (**b**) False alarm probability under *un* = 0 dB; (**c**) Detection probability under *un* = 1 dB; (**d**) False alarm probability under *un* = 1 dB.

Figure 9 illustrates the interrelationship among detection probability, false alarm probability, and SNR in the presence of noise uncertainty. The simulation parameters are set as $P_{f}=0.01$, M=32, $\gamma_{G}=-3\;\mathrm{dB}$, N=1000. From Figure 9a,c, it can be seen that the detection probabilities of the SemiBlind-EM and GLRT decrease with the increase in noise uncertainty. Compared to the two semi-blind methods, the proposed WEMJD has a more stable detection probability under noise uncertainties as it has real-time noise variance estimation capabilities. For the ideal detection LRT, the real-time variation of the known noise variance is considered, so the performance of the LRT is not affected by the noise uncertainty.

It may be noted that the false alarm probability of the proposed method fluctuates around the set value as the global average SNR is varied, which is shown in Figure 9b. When the SNR is greater than −0.5 dB, the actual false alarm probability of WEMJD is less than the set value of 0.01, conversely, when SNR<−0.5 dB, the actual false alarm probability exceeds 0.01. The observed phenomenon can be attributed to the fact that at high SNR, the noise variance estimates increase, causing the SNR estimate in (31) to descend. Consequently, the weighting coefficient in (37) of each user decreases, which will lead to a lower false alarm probability compared to the predetermined threshold value. On the contrary, the $P_{f}$ will become higher at a smaller SNR. For safety considerations, we adopted $\sigma_{n\max}^{2}$ to obtain a lower decision threshold. Therefore, in the presence of noise uncertainty, the actual false alarm probability of the SemiBlind-EM and GLRT is less than 0.01 in Figure 9d. Moreover, by comparing Figure 9b,d, it can be seen that the false alarm probability of WEMJD has increased as noise uncertainty exists. Comparing Figure 9a,c, this increase in false alarm probability can be seen as sacrificing a certain level of false alarm probability to maintain the detection probability.

## 5. Conclusions

As it is often difficult to obtain accurate wireless environment information in real-world scenarios, the estimation and detection methods of cooperative spectrum sensing in dynamic environments are studied in this paper. Based on the LRT theory, it is required to estimate the noise variance, the signal-to-noise ratio, and the channel prior occupancy in the blind scenario with varied channel conditions. A lifting wavelet-assisted EM joint estimation and detection algorithm is proposed to estimate multiple parameters and achieve full-blind detection. Moreover, the lifting wavelet technique is applied in noise variance estimation to improve detection probability and convergence speed. At the same time, a stream learning method is used in estimating SNR and channel prior occupancy rate to fit the dynamic environment. The simulation results demonstrate that the proposed full-blind method can achieve comparable detection performance to the semi-blind EM method. It can adapt to mobile scenarios where the SNR varies over time and exhibit a certain robustness against noise uncertainty.

## Figures and Tables

**Table 1:** The main parameters used in the analysis.

Index	Description
${ℋ}_{1}$	The hypothesis that determines the presence of the PU signal
${ℋ}_{0}$	The hypothesis that determines the absence of the PU signal
z	The channel state/The presence or absence of PU signals.
x	The signal of PU
$h_{j}$	The channel gain from PU to *j*-th SU
$w_{j}$	Noise signal received at *j*-th SU
M	Number of sampling points
J	Number of SU
P	The transmission power of the PU signal
$\sigma_{j}^{2}$	The variance of the noise received at *j*-th SU
${\tilde{\sigma }}_{}^{2}$	The noise variance for all SUs
$\gamma_{j}$	The SNR of the received signal at *j*-th SU
$\tilde{\gamma }$	The SNR of the received signal for all SUs
$T_{j}$	The local detection statistic at *j*-th SU
$\tilde{T}$	Local detection statistics for the entire network
$\pi_{{ℋ}_{0}}$	The prior probability that the PU signal is absent
$\pi_{{ℋ}_{1}}$	The prior probability that the PU signal is present
$\mu_{LRT}$	The threshold for the likelihood ratio test
$\mu_{WEMJD}$	The threshold for the lifting wavelet-assisted EM joint estimation and detection
$\theta_{u}$	The unknown parameter vector
$Q_{j,n}({ℋ}_{1})$	The posterior probability of the presence of the PU signal at the *j*-th SU in *n*-th round detection
$Q_{j,n}({ℋ}_{0})$	The posterior probability of the absence of the PU signal at the *j*-th SU in *n*-th round detection
E[⋅]	Expectation operation
var[⋅]	Variance operation
p	The number of levels of lifting wavelet decomposition.
$w_{p}^{}$	The high-frequency coefficients at the *p*-th level of wavelet decomposition
$c_{p}^{}$	The low-frequency coefficients at the *p*-th level of wavelet decomposition
u	The learning rate
S	The prediction operator
U	The update operator
$a_{j,n}^{*}(z)$	The predicted value of $Q_{j,n}^{*}(z)T_{j,n}$ after learning at *j*-th SU in *n*-th round detection
$b_{j,n}^{*}(z)$	The predicted value of $Q_{j,n}^{*}(z)$ after learning at *j*-th SU in *n*-th round detection
$P_{f}$	False alarm probability
$P_{d}$	Detection probability
T	The random variables of chi-squared-type mixtures
$\psi_{j}$	The weighting coefficient of $T_{j}$
$K_{l}(T)$	The *l*-th cumulant of T

## Data Availability

Not applicable.

## Appendix A

The solution of the estimates involves variable optimization problems with an inequality constraint. Take the estimation of SNR as an example. The optimization problem can be described as

(A1) $$\begin{array}{l} \gamma_{j,n}^{*}=\underset{\gamma_{j,n}^{}\geq 0}{\arg\max}\Lambda \left(\theta_{n},\pi_{z,n}\right)\\ \underset{\gamma_{j,n}^{}\geq 0}{\;\;\;\;\;\;=\arg\min}\left(-\Lambda \left(\theta_{n},\pi_{z,n}\right)\right) \end{array}$$

Take the derivative of the $-\Lambda \left(\theta_{n},\pi_{z,n}\right)$ with respect to $\gamma_{j,n}^{}$, we have:

(A2) $$-\frac{\partial \Lambda \left(\theta_{n},\pi_{z,n}\right)}{\partial \gamma_{j,n}^{}}=Q_{j,n-1}^{}({ℋ}_{1})\left(\frac{M}{1+\gamma_{j,n}^{}}-\frac{T_{j,n}^{}}{\sigma^{2}{}_{j,n}^{*}{\left(1+\gamma_{j,n}^{}\right)}^{2}}\right)$$

The Karush-Kuhn-Tucker (KKT) conditions are

(A3) $$Q_{j,n-1}^{}({ℋ}_{1})\left(M\sigma^{2}{}_{j,n}^{*}\left(1+\gamma_{j,n}^{}\right)-T_{j,n}^{}\right)-\lambda \sigma_{j,n}^{2*}{\left(1+\gamma_{j,n}\right)}^{2}=0,\;\;\;\;\lambda \gamma_{j,n}=0,\;\;\;\;\lambda \geq 0,\;\;\;-\gamma_{j,n}^{}\leq 0$$

where λ is the Lagrange multiplier.

When $\gamma_{j,n}^{}>0$ the constraints are inactive, so λ=0, the KKT conditions reduce to

(A4) $$Q_{j,n-1}^{}({ℋ}_{1})\left(M\sigma^{2}{}_{j,n}^{*}\left(1+\gamma_{j,n}^{}\right)-T_{j,n}^{}\right)=0,\;\;\;\;\gamma_{j,n}^{}>0$$

so we obtain $\gamma_{j,n}^{*}=\frac{T_{j,n}}{M\sigma^{2}{}_{j,n}^{*}}-1$, and the inequality requires $T_{j,n}>M\sigma^{2}{}_{j,n}^{*}$.

When $\gamma_{j,n}^{}=0$ the constraints are active, the KKT conditions reduce to

(A5) $$Q_{j,n-1}^{}({ℋ}_{1})\left(M\sigma^{2}{}_{j,n}^{*}-T_{j,n}^{}\right)-\lambda \sigma^{2}{}_{j,n}^{*}=0,\;\;\;\;\lambda \geq 0$$

so we obtain $\lambda =\frac{Q_{j,n-1}^{}({ℋ}_{1})\left(M\sigma^{2}{}_{j,n}^{*}-T_{j,n}^{}\right)}{\sigma^{2}{}_{j,n}^{*}}$, and the inequality requires $T_{j,n}<M\sigma^{2}{}_{j,n}^{*}$.

The SNR estimate at *j*-th SU can be written as

(A6) $$\gamma_{j,n}^{*}=\max\left(\frac{T_{j,n}}{M\sigma^{2}{}_{j,n}^{*}}\;-\;1,0\right)$$

## Appendix B

The first three cumulants of T can be calculated using the following formulas:

(A7) $$K_{1}(T)=E(T),\;\;\;K_{2}(T)=\mathrm{var}(T),\;\;\;K_{3}(T)=E{\left(T-ET\right)}^{3}\;\;$$

According to (10), the global statistic T can be written as

(A8) $$T=\sum_{j=1}^{J}\frac{\gamma_{j}}{\sigma_{j}^{2}(1+\gamma_{j})}T_{j}$$

Since at FC, the calculation of the weighting coefficient uses estimates of the SNR and noise variance, the actual global statistic is

(A9) $$T=\sum_{j=1}^{J}\frac{\gamma_{j}^{*}}{\sigma_{j}^{2*}(1+\gamma_{j}^{*})}T_{j}$$

where $T_{j}$ is the practical energy of the received signal, so $E[T_{j}]=M\sigma_{j}^{2}$ and $\mathrm{var}[T_{j}]=2M\sigma_{j}^{4}$, then each cumulative quantity can be calculated from (A7):

(A10) $$K_{1}(T)=M\sum_{j=1}^{J}\frac{\gamma_{j}^{*}\sigma_{j}^{2}}{\sigma_{j}^{2*}(1+\gamma_{j}^{*})},\;\;\;K_{2}(T)=2M{\sum_{j=1}^{J}\left(\frac{\gamma_{j}^{*}\sigma_{j}^{2}}{\sigma_{j}^{2*}(1+\gamma_{j}^{*})}\right)}^{2},\;\;\;K_{3}(T)=8M{\sum_{j=1}^{J}\left(\frac{\gamma_{j}^{*}\sigma_{j}^{2}}{\sigma_{j}^{2*}(1+\gamma_{j}^{*})}\right)}^{3}\;\;$$

In full-blind detection, we treat the estimate as the true value, so the above formula becomes

(A11) $$K_{1}(T)=M\sum_{j=1}^{J}\psi_{j},\;\;\;K_{2}(T)=2M\sum_{j=1}^{J}\psi_{j}^{2},\;\;\;K_{3}(T)=8M\sum_{j=1}^{J}\psi_{j}^{3}\;$$

where $\psi_{j}=\gamma_{j}^{*}/\left(1+\gamma_{j}^{*}\right)$.

## Appendix C

The cumulative distribution function (CDF) of T is defined as

(A12) $$F_{T}(t)=P(T<t)=\int_{-\infty }^{t}f_{T}(t)dt(-\infty <t<\infty )$$

when $z={ℋ}_{0}$, the detection threshold is μ, then the false alarm probability can be expressed as

(A13) $$P_{f}=P(T>\mu )=\int_{\mu }^{\infty }f_{T}(t)dt=1-F_{T}(\mu )$$

In constant false alarm rate (CFAR) detection the threshold can be obtained by (A14)

(A14) $$\mu =F_{T}^{-1}(1-P_{fa})$$

Here we use the random variable of the form R=αX+β, $X∼Ga\left(\frac{d}{2},2\right)$ to approximate the distribution of T, therefore

(A15) $$P_{f}=P(X>\frac{\mu -\beta }{\alpha })=\int_{(\mu -\beta )/\alpha }^{\infty }f_{Ga}(x)dx$$

(A16) $$\mu =\alpha F_{{}_{Ga}}^{{}^{-1}}\left(1-P_{f}\right)+\beta$$
